# Safe and Effective Treatment of Intracranial Infantile Hemangiomas with Beta-Blockers

**DOI:** 10.3390/pediatric13030043

**Published:** 2021-07-01

**Authors:** Aoife Naughton, Ariel Yuhan Ong, Goran Darius Hildebrand

**Affiliations:** Oxford Eye Hospital, Oxford University Hospitals NHS Foundation Trust, Headley Way, Headington, Oxford OX3 9DU, UK; yuhan.ong@ouh.nhs.uk (A.Y.O.); darius.hildebrand@ouh.nhs.uk (G.D.H.)

**Keywords:** intracranial infantile hemangioma, beta-blockers, propranolol, timolol

## Abstract

Infantile hemangiomas are common benign vascular tumors but are rarely found in an intracranial location. Our literature review identified 41 reported cases. There is no general consensus on management of these rare lesions and until recently, treatment was limited to surgery or pharmacological management with steroids or interferon. Although beta-blockers have been widely prescribed in the treatment of cutaneous infantile hemangiomas since 2008, their use in the treatment of intracranial infantile hemangiomas has been minimal. We present a case of infantile hemangioma affecting the right orbit, associated with intracranial extension, causing intermittent right facial nerve palsy. The patient achieved an excellent outcome following combined treatment with oral propranolol and topical timolol maleate 0.5%, with complete regression of the lesion by 4 months. We conclude that beta-blockers are a safe and effective treatment of intracranial infantile hemangiomas and can be employed as first-line management of these lesions.

## 1. Introduction

Infantile hemangiomas are common benign vascular tumors, occurring at birth or early infancy, with an estimated incidence of 1–5% [1]. Risk factors for development of infantile hemangioma include Caucasian ethnicity, low birth weight, and female sex (female to male ratio of 2.4:1) [2,3]. They can occur in any organ system but have a predilection for the skin and soft tissues of the head and neck. Intracranial infantile hemangiomas, however, are very rare entities.

Although histologically benign, infantile hemangiomas have a capability for rapid growth. They are characterized by a rapid proliferative phase occurring in the first year of life [4,5]. Most hemangioma growth occurs in the first 5 months, at which point 80% of the final size has often been reached, typically reaching maximal size by nine months [4]. This is followed by a gradual involutional phase, whereby 90% of lesions have spontaneously involuted by five years of age [4,5]. Treatment is therefore not required for most lesions. However, rapid intracranial growth may cause severe neurological complications and thus often requires intervention.

This report was reviewed by the Oxford University Hospitals’ ethics team and formal ethical approval was deemed not to be required. We performed a literature review using Pubmed, searching for keywords “intracranial infantile hemangioma/s”, and identified 41 reported cases (Table 1) [6,7,8,9,10,11,12,13,14,15,16,17,18,19,20,21,22,23,24,25,26,27,28,29,30,31]. There was no uniform treatment approach observed. In some cases of small asymptomatic lesions, watchful waiting was indicated. Some patients were treated surgically [9,12,14,17,19,22,24,26,27]. Others received pharmacological therapy with oral prednisolone [7,10,15,23,25], intralesional triamcinolone [15] interferon alpha [7,25], or thalidomide [11]. Efficacy of these treatments was variable, and in some cases significant side effects were encountered. In 2014, the first cases of successful treatment with propranolol were reported [6].

We present a further case of intracranial infantile hemangioma, where an excellent outcome was achieved with combined topical and systemic beta-blocker treatment for 1 year, with no complications observed and no recurrence following cessation of treatment.

## 2. Case Report

An 8-week-old female infant presented to the Pediatric Ophthalmology clinic with an expanding raised, deep red vascular lesion over the right temporal area which had been present since birth. A few weeks after birth, she developed right proptosis, which continued to progress over the following weeks until review. The proptosis was variable and worse with crying. During this time, her parents also noticed an intermittent right facial nerve palsy with noticeable right facial weakness and an inability to furrow her right brow. She had no past medical history. She was born at 36 + 6 weeks gestation weighing 2.21 kg, following an uncomplicated twin pregnancy.

**Figure 1:**
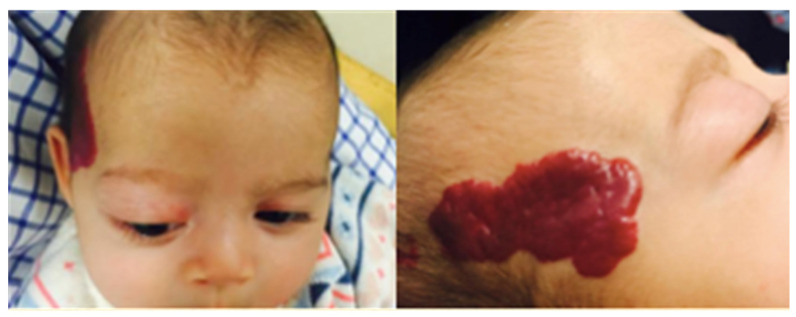
Infant on presentation. Large segmental plaque capillary hemangioma overlying the right temporal area. Marked proptosis with mild lagophthalmos.

**Figure 2:**
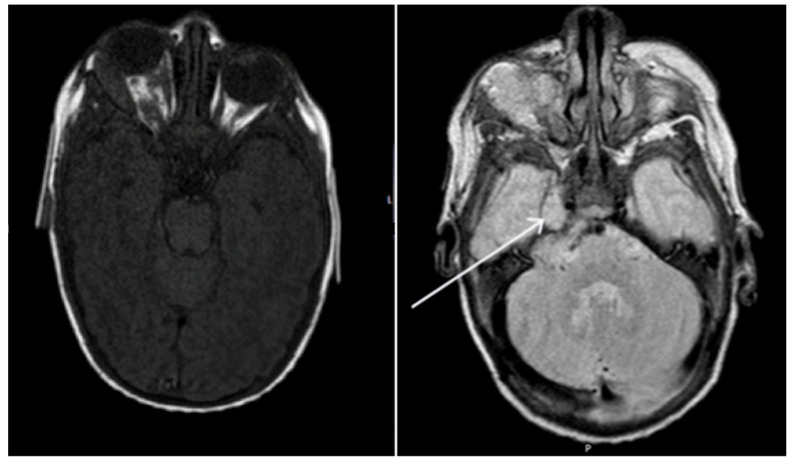
Axial T1 weighted and T2 weighted TSE brain magnetic resonance imaging of patient on presentation. Extensive right orbital lesion with marked proptosis. Extension of the lesion intracranially, with involvement of right Meckel’s cave (pointer) and the cerebellar pontine angle.

On examination, there was a large segmental plaque capillary hemangioma overlying her right temporal area, which was easily compressible. The right eye showed marked proptosis with inability to close the eyelids completely (Figure 1). There was a secondary abduction deficit and right hypoglobus. There was no papilledema on fundal examination. Cycloplegic refraction revealed significant astigmatism in the right eye (+6.00/−3.00 × 40 OD, +5.00 DS OS) as a presumed mass effect of the orbital lesion. Urgent same day MRI imaging was arranged which showed an extensive intra- and extraconal lesion within the right orbit (Figure 2). The lesion was located predominantly within the superolateral compartment of the orbit, extending medially and posteriorly to the level of the optic foramen. The T2-weighted sequence showed that the lesion extended intracranially through the superior orbital fissure, with involvement of right Meckel’s cave and the cerebellopontine angle. The lesion was hyperintense on T2 weighting and relatively isointense on T1 weighting (Figure 2). These characteristics were identical to the cutaneous lesion overlying the right frontal bone. Overall, findings were most consistent with a diagnosis of infantile hemangioma. Due to the segmental plaque presentation and intracranial location, the infant was investigated for PHACE syndrome, but no other clinical or radiological features were found.

After consultation with the Neurology and Dermatology teams, she was commenced on combined topical and systemic beta-blocker treatment. Topical timolol maleate 0.5% (3 drops three times daily) painted onto the periocular area was started in view of the risk of amblyopia. Admission was arranged for commencement of oral propranolol 1 mg/kg in two divided doses. ECG was performed and was normal. Monitoring with regular pulse rate and blood pressure was performed at baseline and at 1 h and 2 h after treatment initiation. These parameters remained within normal limits. Propranolol was well tolerated, and the dose was increased to 2 mg/kg in the second week. Hypoglycemia may be the most common serious complication in children treated with propranolol for IH [32]. The patient’s parents were counselled to ensure a minimum of 6 h between doses, to recognise the symptoms of hypoglycemia, hypotension or bradycardia. The parents were also instructed to ensure that the patient was fed regularly and to avoid prolonged fasts. Infants <6 weeks should be fed at least every 4 h, between 6 weeks and 4 months of age should be fed at least every 5 h, and >4 months of age should be fed at least 6 to 8 h. They were also advised to discontinue propranolol and topical timolol during intercurrent illness, especially in the setting of restricted oral intake.

Occlusion therapy to the left eye was commenced to treat early right amblyopia secondary to anisometropia. Lubricants were prescribed to prevent exposure keratopathy.

**Figure 3:**
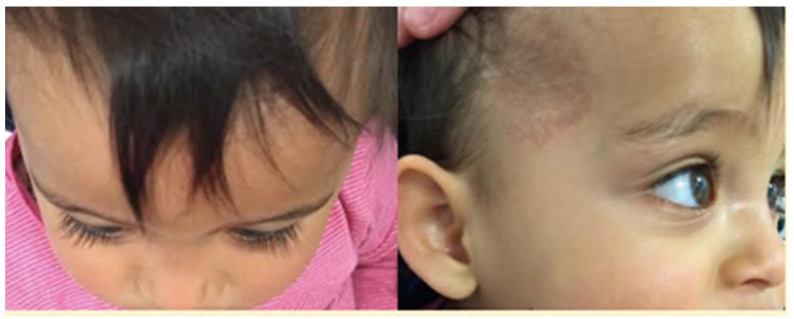
Four months after commencement of treatment. Complete resolution of proptosis. Skin hemangioma flat and pale.

**Figure 4:**
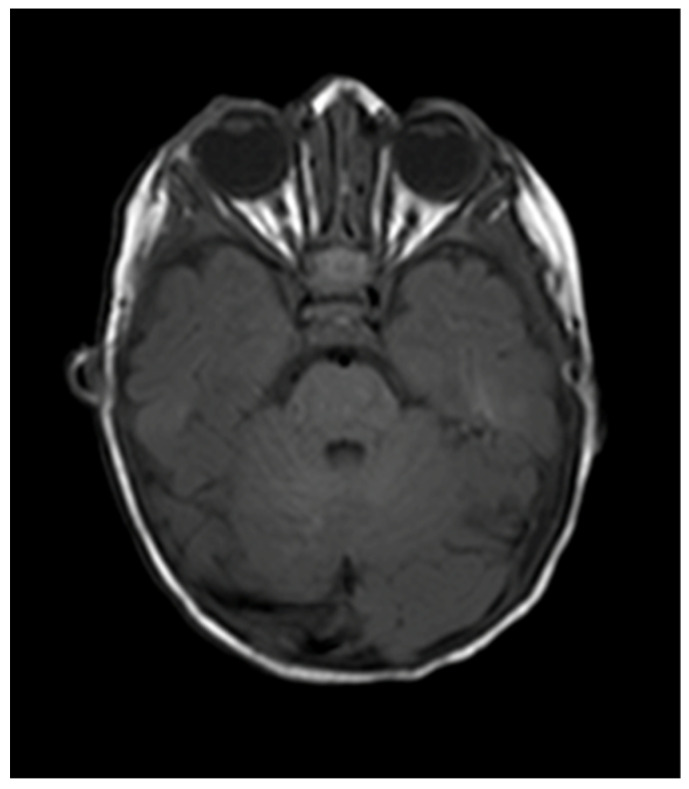
MRI 4 months following commencement of treatment with beta blockers. Total resolution of the orbital hemangioma and of the associated intracranial extension.

A review was arranged nine days after commencement of treatment, at which point the proptosis had started to improve. Three weeks later, the proptosis had reduced significantly, with a marked improvement in the position of the globe and the extraocular movements. At four months of follow-up, the proptosis had completely resolved (Figure 3) and the eye movements were now full. The right temporal skin plaque was now flat and pale. Topical timolol was discontinued at this point, as the vision appeared equal with no evidence of amblyopia. MRI head with gadolinium confirmed total resolution of the right proptosis with no evidence of any residual disease within the orbit (Figure 4). There was also total resolution of the intracranial component of the hemangioma. MRI angiogram confirmed normal appearances of the anterior and posterior cerebral circulation, with no evidence suggestive of PHACE syndrome.

Oral propranolol was continued to complete a 12-month course. No recurrence was observed following treatment cessation.

## 3. Discussion

We identified 41 cases of reported intracranial infantile hemangioma in the literature (Table 1) [6,7,8,9,10,11,12,13,14,15,16,17,18,19,20,21,22,23,24,25,26,27,28,29,30,31]. The majority of patients were female (68%). Mean age at presentation was 3.7 months (range 0–36 months). Most cases tend to occur in association with periorbital hemangiomas, PHACE syndrome, or diffuse neonatal hemangiomatosis. Presenting symptoms were diverse. Over half of patients had no neurological or neuro-ophthalmological signs or symptoms, and their lesions were identified incidentally on imaging of an extracranial lesion. We therefore hypothesize that the true incidence may be underreported. The 20 patients (49%) who did have neurological signs or symptoms had a range of presentations, including seizures, ataxia, central hypotonia, hydrocephalus, enlarged head circumference, and cranial nerve palsy. One patient suffered an arterial ischemic stroke [13]. One infant died following a subarachnoid hemorrhage at the age of 6 weeks [18]

Due to their rarity, there is currently no consensus on the optimal, evidence-based treatment of intracranial infantile hemangiomas. We found that some small asymptomatic lesions were managed with observation alone [13,20,21,23,31], and the hemangiomas spontaneously regressed in some cases [23,31]. Ten patients underwent surgical treatment [9,12,14,17,19,22,24,26,27,30]. In many cases, urgent surgery was necessitated by signs of raised intracranial pressure, or where the preoperative diagnosis was unclear and a more sinister process had been suspected. Surgical resection is associated with a high risk of hemorrhagic complications due to the extensive vascularity of hemangiomas, and alternative treatment options should be considered where possible.

Steroids were the most commonly prescribed pharmacological therapy, with oral prednisolone being the mainstay of treatment [7,10,15,23,25]. Infantile hemangiomas have a variable response to steroid therapy, with one retrospective study reporting regression in one-third, stabilization of growth in another third, and minimal to no response in the final third of lesions located in any organ system [33]. Adverse effects are common, and include irritability, sleep disturbance, adrenal suppression, immunosuppression, hypertension, bone demineralization, and growth retardation [29]. In two cases, intralesional triamcinolone was administered [15], which achieved a reduction in lesion size.

Interferon alpha is an inhibitor of angiogenesis, administered as a daily subcutaneous injection, and has also been used successfully for the treatment of infantile hemangioma [34]. Interferon alpha was used in four patients in our review [7,25], either as primary treatment or following failed corticosteroid therapy. All cases reported a clinical response with reduction in hemangioma size. However, interferon treatment is associated with the risk of significant side effects. Transient neutropenia and liver enzyme abnormalities may develop. Spastic diplegia, irreversible in some cases, has been reported. Consequently, its use is not routinely recommended.

Frei-Jones et al. reported successful treatment of a large unresectable intracranial hemangioma with thalidomide [11]. Treatment did not appear to arrest growth of the lesion initially—despite commencing thalidomide at 35 days of age, its size had increased to 307% of its presenting size at 5 months of age.

In 2008, Léauté-Labrèze et al. described their serendipitous observation that oral propranolol [35], a nonselective blocker of β-adrenergic receptors, was effective and well tolerated in the management of infantile hemangiomas. The mechanism of action likely involves several processes, including vasoconstriction, inhibition of angiogenesis, and stimulation of apoptosis [36,37]. Since then, propranolol has become an increasingly popular treatment for cutaneous infantile hemangiomas, as it is considered to have a better adverse effect profile compared to other systemic therapies. The most commonly reported adverse effects were sleep disturbance and coolness of the distal extremities [38]. Cardiac side effects including bradycardia and hypotension may be encountered; however both are generally asymptomatic and do not require intervention [38]. Less commonly, propranolol can induce hypoglycemia [32]. The risk of this can be minimized by concurrent administration with feedings and withholding doses if oral intake is compromised.

Five cases of intracranial infantile hemangioma successfully treated with oral propranolol have been reported since then. In four cases, there was complete resolution of the intracranial hemangioma [6,8,16], and there was a reduction in lesion size in the final case [28]. No adverse effects were encountered.

We present a rare case of a capillary hemangioma with intracranial and orbital locations, causing a right facial nerve palsy. The patient achieved an excellent outcome following combined treatment with oral propranolol and topical timolol maleate 0.5%, with complete regression of the lesion within 4 months. The patient did not develop any further neurological or neuro-ophthalmic complications, and her amblyopia resolved. Due to their rarity, large scale studies to confirm treatment efficiacy for intracranial infantile hemangiomas are difficult to perform. Our case adds to the small body of evidence that beta blockers are a safe and effective treatment of intracranial infantile hemangiomas and can be employed as first-line management of these lesions.

## 4. Conclusions

Intracranial infantile hemangiomas are rare entities, with varied reported management strategies. This case and review add to the growing body of evidence that beta-blockers are a safe and effective treatment of intracranial infantile hemangiomas and can be employed as first-line management.

## Figures and Tables

**Table 1:** Cases of intracranial infantile hemangiomas.

Author, Year	Age at Presentation, Gender	Neurological or Ophthalmic Complaint	Intracranial Location of Hemangioma	Treatment	Outcome
Willing et al., 1993	17 months, male	Focal seizures, mild developmental delay	Right temporal dura	Surgical excision	Resolution
Bar-Sever et al., 1994	2 weeks, female	Nil	Right middle fossa, extending into the right orbit and suprasellar cistern	Oral prednisolone (for 2 months—no response) Subsequent interferon	Marked reduction with interferon treatment
Tortori et al., 1999	1 month, female	Nil	Right uncohippocampal	Observation	Resolution
	1.5 months, female	Nil	Left CPA, leptomeningeal enhancement at cerebellar surface	Observation	Resolution
	18 months, female	Nil at presentation, developed ataxia	Right CPA, hypothalamus	Systemic steroids, endovascular treatment with contour particles	Unchanged following steroids. Partial resolution following endovascular intervention
	1 month, female	Nil	Left CPA, persistent trigeminal artery	Systemic steroids	Lost to follow-up
Poetke et al., 2002	10 weeks, male	Nil	CPA, leptomeningeal enhancement on cerebellar surface	Nil	Unknown
Le Bihannic et al., 2005	6 weeks, male	Vomiting, disturbance in consciousness, seizures	Anterior choroidal artery, right temporal lobe	Nil	Intracranial haemorrhage, death
Ersoy et al., 2005	8 months, female	Nil	Lateral medullary cistern, IAC, fourth ventricle	Oral prednisolone	Marked reduction in lesion size
Karikari et al., 2006	3 months, male	Central hypotonia	Fourth ventricle, left CPA	Surgical resection	Resolution
Judd et al., 2007	3 weeks, female	Nil	IAC/CPA,	Oral prednisolone	Resolution
	3 weeks, female	Right facial paresis	IAC/CPA, fourth ventricle	Oral prednisolone	Resolution
	6 weeks, female	Nil	IAC/CPA	Intralesional triamcinolone	Resolution
	8 weeks, female	Nil	IAC, Meckel’s cave, cavernous sinus	Intralesional triamcinolone	Resolution
Poindexter et al., 2007	2.5 months, female	Reduced truncal tone	Left IAC	Observation	Partial involution, developmental delay, diffuse hypotonia
Daenekindt et al., 2008	7 weeks, male	Enlarged head circumference	Right temporal fossa	Biopsy, Endovascular embolization and surgical resection	Resolution
Frei-Jones et al., 2008	Newborn, female	Left CNVII palsy, left sensorineural hearing loss	Middle cranial fossa, temporal bone, posterior fossa	Biopsy, Thalidomide	Partial Resolution
Heyer et al., 2008	6 months, female	Nil	Left IAC	Observation	Unchanged
Uyama et al., 2008	4 months, female	Hydrocephalus	Left cerebellar hemisphere	Neuroendoscopic fenestration of cysts, Surgical resection of lesion	Resolution
Viswanathan et al., 2009	3 weeks, female	Hydrocephalus	Quad plate cistern, pineal region, left CPA	Corticosteroids	Reduction in lesion size
	9 weeks, female	Nil	Left cavernous sinus, Meckel’s cave, IAC	Corticosteroids	Lost to follow-up
	4 months, female	Hydrocephalus	Fourth ventricle, left IAC, CPA	Corticosteroids	Reduction in lesion size
	3 months, female	Left ptosis	Fourth ventricle, left foramen of Luschka, quad plate cistern	Interferon, OK432, subsequent corticosteroids	Reduction in lesion size
	7 weeks, female	Right proptosis	Right temporal fossa, cavernous sinus, Meckel’s cave, sella, quad plate cisterns	Corticosteroids	Reduction in lesion size
	7 weeks, male	Nil	Fourth ventricle, right foramen of Luschka, IAC		Reduction in lesion size
	3 weeks, male	Nil	Right CPA, foramen of Luschka, fourth ventricle	Corticosteroids, Interferon	Minimal response to corticosteroids, reduction in lesion size with Interferon
	Infancy, female	Nil	Peri-mesencephalic cistern, sella, cavernous sinus, left CPA	Interferon	Reduction in lesion size
	3 months, female	Nil at presentation, subsequent stroke and hydrocephalus	Right cavernous sinus, CPA	Corticosteroids	Reduction in lesion size
Philpott et al., 2012	12 months, female	Enlarged head circumference	Dura of right parietal lobe	Surgical resection	Resolution
Zheng et al., 2012	3 years, male	Somnolence, right CNIII palsy	Middle cranial fossa	Surgical resection	Resolution
Jalloh et al., 2014	2 weeks, Male	Tense anterior fontanelle, enlarging head circumference, seizures	Left middle cranial fossa	Biopsy, Surgical resection	Residual cyst, no recurrence
Benvenisti et al., 2014	4 weeks, female	Nil	Left posterior fossa	Oral propranolol	Reduction in lesion size, maintained at 12 months
Antonov et al., 2015	3 months, female	Nil	Middle cranial fossa, right cavernous sinus, prepontine cistern, infratemporal fossa	Oral propranolol	Resolution
	3 weeks, female	Nil	Right lateral ventricular trigone	Oral propranolol	Resolution
El Rassi et al., 2015	5 weeks, female	Left CN V and VII palsy (PHACE syndrome)	Left CPA, IAC	Oral propranolol	Improvement in facial lesion, status of intracranial hemangioma not described
Cavalheiro et al., 2016	33 weeks gestation, male	Nil	Posterior fossa	Oral propranolol	Resolution
Kang et al., 2016	1 month, male	Nil	CPA	Oral propranolol	Resolution
Shakir et al., 2016	2 weeks, female	Hydrocephalus	Posterior fossa	Surgical resection	Resolution Post operative enlarging head circumference requiring VP shunt
Dalsin et al., 2016	37 weeks gestation, female	Diagnosed on antenatal ultrasound	Left middle cranial fossa	Surgical resection	Resolution, no neurological deficits
Haine et al., 2017	3 weeks, male	Symptoms of raised ICP	Posterior fossa	Surgical decompression Oral prednisolone	Resolution on imaging
Friedland et al., 2017	1 week, male	Nil	Not specified	Observation	Spontaneous resolution
Naughton et al., 2020 (this paper)	6 weeks, female	Right CNVII palsy	Right orbit, right CPA and Meckel’s cave	Oral propranolol and topical timolol maleate 0.5%	Resolution

## Further Information

The case was presented as a poster at the European Paediatric Ophthalmological Society (EPOS) Annual Meeting 2019, Riga.
